# Evaluation of Bone Mineral Density in Children with Acute Lymphoblastic Leukemia (ALL) and Non-Hodgkin's Lymphoma (NHL): Chemotherapy with/without Radiotherapy

**Published:** 2016-07-01

**Authors:** Ali Ghassemi, Abdollah Banihashem, Nosrate Ghaemi, Saghi Elmi, Reza Erfani Sayyar, Sam Elmi, Habibollah Esmaeili

**Affiliations:** 1 Department of Pediatric, Hematology and Oncology and Stem Cell Transplantation, Mashhad University of Medical Sciences, Mashhad, Iran; 2 Department of Pediatric, Hematology and Oncology, Mashhad University of Medical Sciences, Mashhad, Iran; 3 Department of Pediatric, Endocrine and Metabolism, Mashhad University of Medical Sciences, Mashhad, Iran; 4 Department of Pediatric, Intensive Care Unit (PICU), Mashhad University of Medical Sciences, Mashhad, Iran; 5 Department of Intensive, Care Unit (ICU), Mashhad University of Medical Sciences, Mashhad, Iran; 6 Department of Pediatrics, Mashhad University of Medical Sciences, Mashhad, Iran; 7 Department of Biostatistics and Epidemiology and Health Sciences Center, Mashhad University of Medical Sciences, Mashhad, Iran

**Keywords:** Bone mineral density, Radiotherapy, Chemotherapy, Acute lymphoblastic leukemia (ALL), Non-Hodgkin's lymphoma (NHL)

## Abstract

**Background:** Acute lymphoblastic leukemia (ALL) and non-Hodgkin's lymphoma (NHL) are the most common malignancies in children and adolescents. Therapies such as corticosteroids, cytotoxic and radiotherapy will have harmful effect on bone mineral density (BMD) which can lead to increased possibility of osteoporosis and pathological fractures.

**Subjects and methods:** This 3-year cross-sectional study was performed in 50 children with ALL (n=25) and NHL (n=25) at Dr. Sheikh Children's Hospital in Mashhad. Half the patients received chemotherapy alone (n=25), while the other half received chemotherapy plus radiotherapy (n=25). We assessed them in the remission phase by DEXA bone mineral densitometry at the lumbar spine and femoral neck (hip). The survey results were adjusted in accordance with age, height, sex and Body Mass Index.

**Results** **:** The mean age was 3.93± 8.28 years. There was no significant difference in bone biomarkers (Ca, P, ALP, PTH) among ALL, NHL and also the two treatment groups. Children with ALL had lower density at the hip and lumbar spine (p-value<0.001 and p-value=0.018, respectively). Among the total of 50 patients, 3 patients had normal results for detected hip BMD (6%), while 14 (28%) had osteopenia and 33 had osteoporosis (66%). Only one patient had normal BMD among all the patients who received chemotherapy plus radiotherapy, whereas 2 patients had normal BMD with just chemotherapy treatment.

**Conclusion** **:** Given that 94% of our patients had abnormal bone density, it seems to be crucial to pay more attention to the metabolic status and BMD in children with cancer.

## Introduction

 As the incidence rate of cancer in patients younger than 19 years is not very high, the 5-year survival has increased recently. However, cancer remains one of the most prominent leading cause of death in children between 1 to 14 years in many countries.^1^ Acute lymphoblastic leukemia (ALL) and non-Hodgkin's Lymphoma (NHL) are the most common malignancies in children and adolescents.^2^ With recent advances in the diagnosis and treatment, the 5-year survival has progressed for ALL and even more for NHL.^3^ But about 80% of these children who have improved at a young age will develop complications due to treatment in later life. Particularly, most of these disorders remain until adolescence and adulthood. Therapies such as corticosteroids, cytotoxics and radiotherapy will have harmful effect on bone mineral density (BMD) and can increase the possibility of osteoporosis and pathological fractures due to loss of bone density.^4^^,^^5^ Accurate statistics is not available on the incidence and severity of bone loss.^6^ Other risk factors which can cause a long-term decline in bone density include cancer itself, poor nutrition, decreased exposure to sunlight and low levels of physical activity during treatment.^7^^,^^8^ Long-term musculoskeletal effects of chemotherapy in cancers may include AVN (avascular necrosis) and a decrease in bone density.^9^^,^^10^ Despite the decrease in bone density after completion of treatment, BMD will be better in the long run during adolescence; however, they are at risk of osteoporosis. Therefore, bone mineral densitometry is recommended to perform in patients at risk of osteoporosis at regular intervals.^4^^,^^11^ Two current standard methods used for BMD assessment are Dual*-*energy x-ray absorptiometry (DEXA) and quantitative CT. DEXA results should be adjusted for age, pubertal stage and height (Z-score in children but not T-score).^12^ It is noteworthy that there are DEXA machines with different brands that provide different results; therefore, only the same model should be used for the patient during routine follow-up.^13^ So, we decided to assess BMD and bone biomarkers in children with ALL and NHL at Dr. Sheikh Children's Hospital in Mashhad.

## SUBJECTS AND METHODS

 This cross-sectional study was performed from 2010 to 2013 at Dr. Sheikh Children's Hospital in Mashhad. Initially, a total of 70 patients were included in the study. Three patients were not willing to participate in the study. Nine patients withdrew from the study*,* 4 patients died during the study period and 4 were excluded due to recurrence. Therefore, this study was eventually conducted in 50 patients. Since the treatment of non-Hodgkin's lymphoma (including all histological types) is identical with acute lymphoblastic leukemia, in order to avoid confounding statistical factors; we ignored details in treatment such as radiation dose, type and amount of drugs used in chemotherapy. In general, patients were divided into two groups: one group received chemotherapy alone (n=25) and the other received chemotherapy combined with radiation therapy (n=25). Then, all of these 50 patients involving two groups were divided into another two groups: ALL and NHL. Each group consisted of 25 patients who were in remission phase after treatment.

Inclusion criteria were: 1) aged 3 to 17 years (Table 1); 2) less than 14 years of age at the time of diagnosis; 3) time interval of 5 years between the completion date of chemotherapy and study entrance date; and 4) the remission phase of the bone marrow (maintenance phase or completed treatment).

Exclusion criteria were: 1) history of secondary malignancy due to previous treatments; 2) congenital disorders like Fanconi anemia, chromosomal syndromes such as Bloom syndrome; 3) history of bone marrow transplantation and 4) bone fracture.

Adopting the pertinent chemotherapy and radiotherapy protocols, hematologic assessment and bone marrow evaluation showed that all patients were in complete remission. Given the demographic information such as age, gender, location, we measured weight by SECA Sinker and the height by stadiometer in standing position. BMI (Body Mass Index) was calculated using the following formula: weight (kg)/height squared (cm^2^).

We assessed all children for bone density by DEXA (Dual energy X-ray Absorptiometry), with no injections and no special preparation. The device used in this study was Hologic discovery. We performed densitometry on the femoral neck (hip) and lumbar spine (L2-L4). The Z-score was calculated regarding age and sex for bone density. Values obtained as Z–Score of hip (H) and the lumbar spine (S). The values were matched and compared for bone density in people of the same sex, age and race via software of densitometry itself. According to WHO (World Health Organization) definition, Z-Score greater than or equal to -1 is called normal bone density, between -1 and –2.5 is called osteopenia, showing reduced bone mineral density and less than or equal to -2.5 is called osteoporosis, showing severe loss in bone mineral density. In all patients, bone biomarkers including levels of calcium (Ca), phosphorus (P), PTH hormone (PTH) and alkaline phosphatase (ALP) in plasma were measured.

We used the t-test for variables with normal distribution and Mann-Whitney test for non-normally distributed variables. Regarding the qualitative variables (name and rank), both the Fisher's exact test and the Chi–square test were used in the two groups of cross-tab. P≤0.05 is considered as a statistically significant difference. The sample size was calculated according to the following formula:^18^


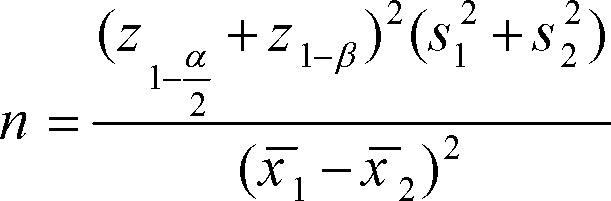


We increased our sample size to 25 patients in each groups to prevent sample loss.

Non-probability sampling technique was simple and purposeful. The practical checklist was used as a data collection method.

## Results

 Among patients with ALL and NHL, 12 and 10 patients were female, respectively (p-value= 0.569). The mean age ± SD (standard deviation) of the patients with ALL was 7.06 ± 3.68 years, while it was 9.50 ± 3.86 in patients with NHL. Totally, mean age ± SD was 8.28 ± 3.93 (range: 3-17 years) in all patients. Table 1 shows the age distribution of the patients in different types of treatment.

Our findings are classified as follows: 1) bone biomarkers 2) densitometry results including quantitative densitometry [Z-score S (lumbar spine) and Z-score H (hip)] and qualitative results of bone mineral densitometry (BMD) based on WHO definition.

With regard to the Tables 2 and 3, there are no significant discrepancies in biomarkers of bone (Ca, P, ALP and PTH) among ALL, NHL and also two study groups with different treatment regimens as the p-value was greater than 0.05. For the PTH and ALP, Mann-Whitney test was used, while the Ca and P were evaluated by the means of T-test. All data were individually adjusted by the age in laboratory reference books and Nelson pediatric reference (2011).

**Table 1:** The age distribution of the patients in different types of treatment

**Type of treatment**	**Chemotherapy alone** **(n=25)**	**Chemotherapy plus radiation** **(n=25)**	**Total** **(n=50)**
**Age (year)**			
Mean ± standard deviation	8.28 ± 4.05	8.28 ± 3.88	8.28 ± 3.93
Minimum	3.00	3.00	3.00
Maximum	17.00	17.00	17.00

**Table 2:** Comparison of bone biomarkers mean and standard deviation of variables in the 2 studied groups

**Variable**	**ALL** **n=25** **Mean and ** **standard deviation**	**NHL** **n=25** **Mean and standard deviation**	**Results**
PTH	50.65 ± 63.73	34.88 ± 9.72	p-value= 0.225 z=-1.23
ALP	489.40 ± 159.80	619.36± 355.52	p-value= 0.099 z=-1.64
Ca	9.24 ± 0.74	9.49 ± 0.70	P-value= 0.232 t=-1.20
P	4.61 ± 0.89	4.40 ± 0.98	P-value= 0.445 t=-1.591

**ALL:** Acute Lymphoblastic Leukemia, **NHL:** Non-Hodgkin's Lymphoma, **PTH:** Parathromone hormone, **ALP:** Alkaline phosphatase, **Ca:** Calcium, **P:** Phosphorus

**Figure 1:**
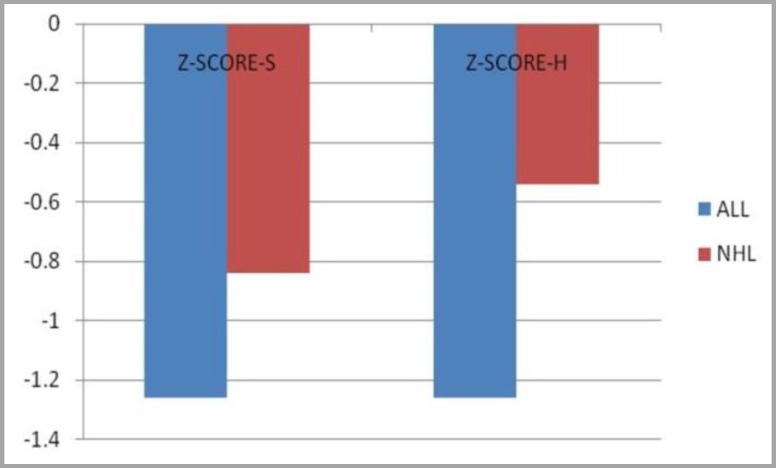
Comparison of the mean z-score bone density at the hip (H) and lumbar spine (S) depending on the type of disease. **ALL:** Acute Lymphoblastic Leukemia, **NHL:** Non-Hodgkin's Lymphoma

**Figure 2:**
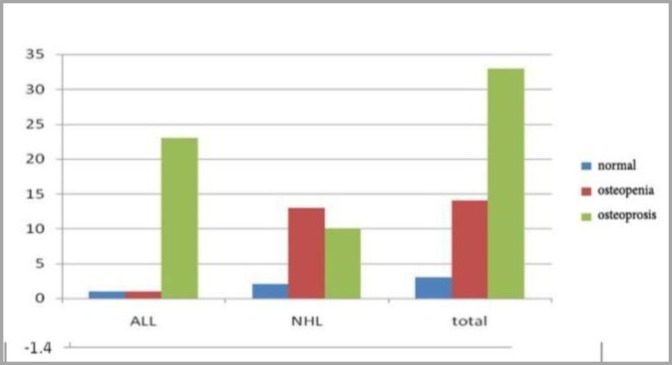
Comparison of BMD at the hip (as defined by WHO) in studied groups. **ALL:** Acute Lymphoblastic Leukemia, **NHL:** Non-Hodgkin's Lymphoma, **BMD:** Bone Mineral Density

In patients with ALL, Z-S core H (hip or femoral neck) was lower than patients with NHL which means bone density at the femoral neck in patients with ALL more decreased clinically. Also, there was statistically significant difference between the two groups (ALL, NHL) regarding the Z-Score-H (p-value =0.04), contrary to the Z-Score-S (lumbar spine L2-L4) (p-value=0.118) (Figure 1). Moreover, there was no statistically significant difference between the two treatment groups (chemotherapy alone or plus radiotherapy) regarding the Z-Score for hip and lumbar spine (p-value was 0.205 and 0.219, respectively). Qualitative results of bone mineral densitometry (BMD) based on WHO definition showed that in patients with ALL (n=25), 23 (46%) had osteoporosis, 1 (2%) had osteopenia and only one case (2%) had normal bone density. In a total of 50 patients, only 3 (6%) had normal bone density at hip site; therefore, most of whom had decreased bone density. Totally, 14 patients (28%) showed osteopenia and 33 (66%) showed osteoporosis. There were statistically significant differences in BMD at the hip between the two groups of patients with ALL and NHL (p-value<0.001). Children with ALL had lower density compared to NHL counterparts (Figure 2).

BMD evaluation at the lumbar spine (L2-L4) showed that none of 25 patients with ALL had normal bone mineral density at the lumbar spine (zero percent), 3 (6%) had osteopenia and 22 (44%) had severe decreased bone density called osteoporosis (Table 4). In the group of patients with NHL (n=25), only 4 patients (8%) had normal lumbar spine bone density, 6 (12%) had osteopenia and 15 (30%) had osteoporosis. Unfortunately, 46 (92%) of 50 patients had decreased bone density in lumbar region. Compared with WHO definition, 9 patients (18%) had osteopenia and 37 (74%) had osteoporosis.

**Table 3:** Compression of bone biomarkers mean and standard deviation of variables in the 2 studied groups with different treatment regimens

**Variable**	**Chemotherapy plus with radiotherapy** **n=25** **Mean and standard deviation**	**Chemotherapy** ** alone** **n=25** **Mean and standard deviation**	**Results**
PTH	50.58 ± 6.31	34.95± 13.70	p-value=0.210 z=1.25
ALP	497.40 ± 181.28	611.36 ± 347.99	p-value=0.281 z=-1.07
Ca	9.25 ± 0.60	9.49 ± 0.83	p-value=0.284 t=-1.16
P	4.51 ± 0.84	4.51 ± 1.02	p-value=0.964 t=0.045

**PTH:** Parathromone hormone, **ALP:** Alkaline phosphatase, **Ca:** Calcium, P: Phosphorus

The Mann-Whitney test showed that 22 (44%) patients with ALL had osteoporosis, compared to NHL group (30%). Although there was no statistically significant difference in lumbar spine between the two groups (p value=0.018), bone densitometry results in ALL group were worse than NHL group (Table 4).

**Table 4:** Comparison of BMD in the lumbar spineL2-L4 (as defined by WHO) between patients with ALL and NHL

**Type of disease**	**ALL** **n ** **(** **%)**	**NHL** **n ** **(** **%)**	**Total** **n ** **(%)**
**BMD in the lumbar spine**			
Normal	0 (0)	4(8.0)	4(8.0)
Osteopenia	3(6.0)	6(12.0)	9(18.0)
Osteoprosis	22 (44.0)	15(30.0)	37(74.0)
Total	25 (50.0)	25 (50.0)	50 (100)
Result Mann–Whitney test	Z=2.36 p value=0.018		

**ALL:** Acute Lymphoblastic Leukemia, **NHL:** Non-Hodgkin's Lymphoma, **BMD:** Bone Mineral Density

BMD in hip (as defined by WHO) was measured and compared between 2 treatment groups:

A) In the group with the combined therapy of radiotherapy with chemotherapy, only one patient had normal BMD, while 24 patients (48% of all the 50 patients) had a reduction in BMD, of whom 14 (28%) had osteoporosis.

B) In the group with chemotherapy alone, 2 patients (4%) had normal bone density at the hip site, while 23 (46%) had decreased bone density.

However, no statistically significant difference was found in BMD at the hip site between these two treatment groups (p-value=0.203) (Table 5).

Moreover, considering the lumbar spine BMD in the 2 aforementioned treatment groups, 22 (44%) and 24 (48%) patients had decreased BMD in the first and second group, respectively. Therefore, no statistically significant difference was found in lumbar spine BMD between the two treatment groups (L2-L4) (p=0.106). Having included a crucial factor called chemotherapy duration in month in our study, we compared it with BMD at the hip (Z-Score H) and at the lumbar spine (Z-Score S) by means of Spearman's rho test and no significant disparity was found (p-value=0.082 and p-value=0.136, respectively) (See Table 6). The result of the Mann-Whitney test (Table 6) showed that there was no significant difference between the type of disease (ALL, NHL) and duration of chemotherapy (p=0.128).

The result of Fisher's exact test showed that there was no significant difference at the hip and lumbar spine between sex and bone density as defined by WHO (p-value was 0.553 and 0.490, respectively). No significant difference was found in gender distribution of patients according to the Z-score for Hip and spine bone (p=0.968 and p=0.614).

## Discussion

 This 3-year cross-sectional study was performed in 50 children with ALL (n=25) and NHL (n=25) at Dr. Sheikh Children's Hospital, Mashhad. Half the patients received (n=25) chemotherapy alone, while the other half received chemotherapy plus radiotherapy (n=25). We assessed them by the means of DEXA bone mineral densitometry (BMD) method at the lumbar spine and neck of femur (hip). We also measured some bone biomarkers including calcium (Ca), phosphorus (P), parathoromone (PTH) and alkaline phosphatase (ALP) in plasma. The mean age was 8.28 ± 3.93 years. There was no significant difference in bone biomarkers (Ca, P, ALP, PTH) among patients with ALL or NHL and also two treatment groups.

**Table 5:** Comparison of BMD in hip (as defined by WHO) between two treatment groups

**Type of ** **treatment**	**Chemotherapy ** **plus with ** **radiotherapy** **n (%)**	**Chemotherapy** **alone** **n (%)**	**Results** **n (%)**
**BMD in the hip**			
Normal	1 (2.0)	2 (4.0)	3 (6.0)
Osteopenia	10 (20.0)	4 (8.0)	14 (28.0)
Osteoporosis	14 (28.0)	19 (38.0)	33 (66.0)
Total	25 (50.0)	25 (50.0)	50 (100.0)
Result Mann–Whitney test	z=1.27 p-value=0.203		

**ALL:** Acute Lymphoblastic Leukemia, **NHL:** Non-Hodgkin's Lymphoma **BMD:** Bone Mineral Density

**Table 6:** Comparison of chemotherapy duration (months) in the study groups in terms of disease

**Type of disease**	**ALL** **n=25**	**NHL** **n=25**
**Chemotherapy** **duration (** **in ** **months)**		
Rank mean and standard deviation	22.40 ± 14.41	28.60 ± 15.65
Result Man–Whitney test	p-value=0.128 z=-1.5	

**ALL:** Acute Lymphoblastic Leukemia, **NHL:** Non-Hodgkin's Lymphoma

The mean age was 8.28 ± 3.93 years. There was no significant difference in bone biomarkers (Ca, P, ALP, PTH) among patients with ALL or NHL and also two treatment groups. Children with ALL had lower density at the hip and lumbar spine (p-value was <0.001 and 0.018, respectively). The result of hip BMD in 50 patients showed that 3 (6%) had normal values, 14 (28%) had osteopenia and 33 (66%) had osteoporosis.

Among patients who received radiotherapy plus chemotherapy, only 1 patient had normal rate of BMD at the hip and 24 (48%) had a BMD reduction at hip*.* Similarly, 22 patients (44%) had decreased BMD amounts at lumbar spine in the pertinent group.

By contrast, in patients who received only chemotherapy, 24 (48%) and 23 (46%) patients showed osteoporosis at hip and lumbar spine, respectively. No significant difference was found in BMD between genders.

In our present study, since bone density in ALL patients is more decreased than patients with lymphoma (NHL), we considered that it can be contributed to the type of disease which needed to be treated with more vigorous chemotherapy in ALL patients.

In a study by Van der SluisIm et al. in the Netherlands, BMD in 61 children with ALL who received dexamethasone-based chemotherapy was examined. They evaluated total body and lumbar spine BMD by DEXA method at time of diagnosis and one year after treatment. They found that BMD at the lumbar spine had decreased severely at the time of diagnosis and then during treatment this ratio remained steadily low. However, total body BMD was normal at baseline, 32 weeks after treatment BMD had a slump in those receiving substantially chemotherapy. Fracture rates in children with ALL were compared with healthy controls which had six times as many fractures as the latter.^14^

Bone density and risk factors in 70 children with ALL were assessed by Gunes AM et al. in Turkey. The survey result showed that 44% of patients had osteoporosis, 41% had osteopenia and remaining had normal BMD. Moreover, some risk factors such as calcium intakes, time interval between completion of chemotherapy and cranial radiotherapy, the total dose of steroids and reduction of physical activity were evaluated. This study showed a positive correlation between daily calcium intake and bone density (p=0.0015).^15^

However, in our study, none of the 25 children with ALL had normal BMD at the lumbar spine. Additionally, at the same region, 3 (6%) patients had osteopenia, while 22 (44%) had osteoporosis. Only one patient had normal values at the hip and 1 had osteopenia. Totally, 23 cases of osteoporosis were reported in our study.

It is noteworthy that lifestyle and conditions of nutritional and racial in Turkey and Iran are equally the same. Therefore, it is worth comparing a Turkish study with our results. In their study, 85% of ALL survivors had reduced bone density, whereas in our study 96% of patients had osteoporosis at the hip.^15^ Meanwhile, all patients had osteoporosis at the lumbar spine, indicating that the bone density in our children with ALL, unfortunately, has been worsened.

Moreover, in Turkey’s survey, levels of ALP, phosphorus, calcium, magnesium, 25-hydroxy vitamin-D and IGF-1 were assessed at the end of treatment in children (n=70) whose IGF 1 and 25-hydroxyvitamin D were reported lower than control group (p=0.033)^15^ Compared with our study included 25 children with ALL, the amounts of Ca, P, PTH and ALP were in normal range.

In another study, AliKasifoglu et al.^18^ have assessed the effect of different protocols of treatment on bone density and bone biomarkers in 36 boys and 23 girls with ALL (mean age of 11.7 years) after completion of chemotherapy since 2005. The first group received conventional dose of prednisolone (CDP) and the second group was treated with high-dose methylprednisolone (HDMP). Finally, there was no significant difference between the two groups in Z-Score of BMD (p=0.73). Also, no significant difference in bone biomarkers was seen. The mean bone mineral density remained the same before and after the maturity. Nevertheless, they concluded that HDMP regimen (compared to CDP) did not deteriorate bone density. They also emphasized that treatment with high-dose corticosteroids in short term after induction therapy-remission will not affect the bone mass, so they suggested it is better to evaluate bone complications 3 years after the treatment accomplishment.^16^ We did not specifically study the corticosteroids.

El-Ziny MA et al. in Egypt evaluated bone density of the vertebrae L2-L4 and hip in 20 children with lymphoma immediately after treatment. They found that 23 (86%) and 21 (81%) of patients had decreased bone density in the hip area and vertebrae L2-L4, respectively, but the follow-up was not done.^17^ The results of this study showed disappointing results in patients with NHL.

In the survey of Benmiloud S et al. 89 children with ALL and NHL were assessed for the long-term effects of treatment on bone density after 5 years of remission in Belgium. Furthermore, patients were divided into 3 categories based on their treatments: A) only chemotherapy; B) combined chemotherapy and radiotherapy; C) BMT/TBI (bone marrow transplantation and total-body radiation).^18^

Bone loss was observed in 44 patients, of whom males were found to have a higher rate. Comparatively, although group A had decreased bone density, group B experienced more reduction rate of bone density at neck of femur since the latter had received additional dexamethasone, especially in the area of the lumbar spine where BMD was more decreased.

Group C patients who underwent radiotherapy and bone marrow transplantation had lower lumbar bone density.^18^

This study was similar to ours in many related aspects. We had 50 patients, of whom 25 had ALL and 25 had NHL. We also considered two treatment groups receiving chemotherapy alone and chemotherapy plus radiotherapy. However, bone marrow transplantation was not performed in our patients. In this study, there was no significant difference at hip and lumbar spine BMD between the two study groups. In contrast to the above study, patients who received radiotherapy and bone marrow transplantation had more escalating BMD. Furthermore, our study showed worse BMD in patients with ALL than those with NHL. In the above study, boys had higher bone density reduction, whereas no significant difference was observed between BMD and gender in our study.

We had some restrictions in our study. Taking into account that the survey on BMD of children with malignancy at Dr Sheikh children’s Hospital in Mashhad was conducted for first time, therefore, some preliminary data were not available. In order to achieve the correct statistical evaluation, we had to omit some variables. Furthermore, in order to preserve our basic moral issues, the study population was reduced to 50 because some of our patients refused to participate, withdrew or died during the study period. Due to the late effects of treatment on bone mineral density of children and adolescents, it seems that patients’ assessment such as height, weight, BMI and BMD at diagnosis and regular intervals is necessary. Long-term follow-up after treatment is recommended to improve their quality of life, either physically or socially.

It seems that modification of diet in patients receiving chemotherapy and supplements such as calcium and vitamin D and age-appropriate activities can help the patient in the process. This study aimed at improving the quality of life and encouraging authorities to plan a comprehensive study including a greater number of patients with complete records of risk factors. On the other hand, according to high prevalence of low bone mineral density in our patients with ALL and lymphoma, it is worth considering the high cost of treatments. Hence, the need of proper insurance supports would be of a crucial importance.

## CONCLUSION

 Given that 94% of our patients had abnormal bone density, it seems to be crucial to pay more attention to the metabolic status and BMD in children with cancer.
